# Prospectus of Genomic Selection and Phenomics in Cereal, Legume and Oilseed Breeding Programs

**DOI:** 10.3389/fgene.2021.829131

**Published:** 2022-01-21

**Authors:** Karansher S. Sandhu, Lance F. Merrick, Sindhuja Sankaran, Zhiwu Zhang, Arron H. Carter

**Affiliations:** ^1^ Department of Crop and Soil Sciences, Washington State University, Pullman, WA, United States; ^2^ Department of Biological System Engineering, Washington State University, Pullman, WA, United States

**Keywords:** genetic gain, genomics, high throughput phenotyping, machine and deep learning, plant breeding, root phenomics

## Abstract

The last decade witnessed an unprecedented increase in the adoption of genomic selection (GS) and phenomics tools in plant breeding programs, especially in major cereal crops. GS has demonstrated the potential for selecting superior genotypes with high precision and accelerating the breeding cycle. Phenomics is a rapidly advancing domain to alleviate phenotyping bottlenecks and explores new large-scale phenotyping and data acquisition methods. In this review, we discuss the lesson learned from GS and phenomics in six self-pollinated crops, primarily focusing on rice, wheat, soybean, common bean, chickpea, and groundnut, and their implementation schemes are discussed after assessing their impact in the breeding programs. Here, the status of the adoption of genomics and phenomics is provided for those crops, with a complete GS overview. GS’s progress until 2020 is discussed in detail, and relevant information and links to the source codes are provided for implementing this technology into plant breeding programs, with most of the examples from wheat breeding programs. Detailed information about various phenotyping tools is provided to strengthen the field of phenomics for a plant breeder in the coming years. Finally, we highlight the benefits of merging genomic selection, phenomics, and machine and deep learning that have resulted in extraordinary results during recent years in wheat, rice, and soybean. Hence, there is a potential for adopting these technologies into crops like the common bean, chickpea, and groundnut. The adoption of phenomics and GS into different breeding programs will accelerate genetic gain that would create an impact on food security, realizing the need to feed an ever-growing population.

## Introduction

Classical plant breeding has evolved considerably during the last century. This can be attributed to the combined action of molecular markers, improved experimental designs, statistical methods, understanding of the concepts of population and quantitative genetics, and integration of other disciplines such as entomology, pathology, soil science, engineering, agronomy, and physiology (Lopes et al., 2012; Ray et al., 2012). The evolution and adoption of all these techniques and tools has pushed the annual genetic gain of grain yield approximately 1% for major cereals like maize (*Zea mays* L.), rice (*Oryzae sativa* L.), and wheat (*Triticum aestivum* L.) (Lopes et al., 2012; Masuka et al., 2017a; Cobb et al., 2019). However, the rate of genetic gain in these crops is insufficient to cope with a 2% annual increase in the human population, which is expected to reach 9.8 billion by 2050 (Ray et al., 2012, 2013). Plant breeders and scientists are under pressure to develop new varieties and crops having higher yield, higher nutritional value, climate resilience, and disease and insect resistance. The solution requires the merging of new techniques like next-generation sequencing, genome-wide association studies, genomic selection, high throughput phenotyping, speed breeding, and CRISPR mediating gene editing with previously used tools and breeder’s skills (Varshney et al., 2021).

Since the 1980s, various molecular marker systems such as restriction fragment length polymorphism (RFLP), amplified fragment length polymorphism (AFLP), randomly amplified fragment polymorphic DNA (RAPD), simple sequence repeats (SSR), and single nucleotide polymorphism (SNP) have been developed and led to the identification of several quantitative trait loci (QTL) by linkage mapping in most crops (Zhu et al., 2008; Buerstmayr et al., 2009). This was further supported by the development of high throughput genotyping tools like diversity array technology (DArT), genotyping by sequencing (GBS), SNP array platform (for instance in wheat, several high-density SNP arrays are available including the Illumina Wheat 9K iSelect, Wheat 15K SNP array, 35K Axiom array developed from an 820K array, 55K SNP array developed from 660 arrays, Illumina 90K iSelect SNP array, and the Axiom wheat 660K SNP array), and next-generation sequencing, all of which provide tremendous amounts of marker information for utilization in mapping studies (Poland and Rife, 2012; Wang et al., 2014; Cui et al., 2017). Linkage mapping started with great hype for deciphering each trait’s genetic architecture and improving traits. This hype was later unrealized and attributed to low mapping resolution, QTL by genotype interaction, QTL by environment interaction, and QTL specific to a particular segregating population. However, there are some success stories utilizing linkage mapping for cultivar development, such as identification of *Sub1* QTL for submergence tolerance in rice, *Fhb1* QTL for providing tolerance to fusarium head blight in wheat, and QTL for providing resistance to cyst nematodes in soybean (*Glycine* max L.) (Concibido et al., 2004; Anderson et al., 2007; Septiningsih et al., 2009).

As the excitement about linkage mapping began to fade in the early 2000s, association mapping emerged as a new technique for studying marker-trait associations (Lander and Botstein, 1989; Breseghello and Sorrells, 2006; Yu et al., 2006). Association mapping has two main advantages over linkage mapping. Firstly, it saves the time, cost, and effort required to create a mapping population, as it uses a collection of germplasm, which can be easily assembled. Secondly, QTL can be mapped with higher resolution due to multiple historical recombination in the germplasm (Korte and Ashley, 2013). Several statistical models were developed, which varied from single locus to multi-locus models and multivariate models, including genotype by environment interaction, dominance, and epistasis components depending upon the associated crop’s nature (Huang et al., 2018; Tibbs Cortes et al., 2021). It was later realized that association mapping suffers from several limitations and has not shown the same potential as linkage mapping. The main reason for its low success was that it detects variants common in the mapping panel and thus has low power for detecting the rare variant. These rare variants could be identified by linkage mapping with segregation of alleles in the mapping population, which will provide higher power to detect rare QTL. Furthermore, several nested association mapping (NAM) and multi-parent advanced generation intercross (MAGIC) populations have been developed in most of the crop species discussed in this review for marker trait associations (MTAs) with high power and resolution during mapping studies (Yu et al., 2008; Diaz et al., 2021; Sandhu et al., 2021d).

By the late 2000s, plant breeders realized that they needed a technique that can not only identify associated QTL, but provides enough information to improve complex quantitative traits, for which previous mapping techniques had failed. Bernardo (Bernardo, 1994) achieved the earliest success for predicting breeding values by replacing pedigree based matrix with a marker based kinship using RFLP markers in maize. The term genomic selection (GS) was first coined in 2001 and uses whole genome-wide markers for predicting genomic-estimated breeding values (GEBVs) of individuals (Meuwissen et al., 2001; Bassi et al., 2015). GS is a technique that is not a design approach to create a cultivar with a specific QTL combination but uses a predictive approach to identify the line with the best breeding values using whole genome wide markers. It uses hundreds to thousands of genome-wide markers and previous years phenotypic data to build the GS model and predict the performance of new lines for quantitative traits (Isidro et al., 2015). If a marker is in linkage disequilibrium (LD) with the associated QTL, it will capture a large proportion of the genetic variance for predicting that trait’s performance. The interest of GS in plant breeding started after it was reported in maize in 2007 (Bernardo and Yu, 2007), and subsequently, several studies followed up utilizing this technique in different crop species (Crossa et al., 2014; Sun et al., 2017b). Plant breeders are rapidly adopting GS for selecting the parents of new crosses, removing poorly performing lines, predicting the performance of lines in untested environments, predicting quantitative traits early in the breeding pipeline (which was previously difficult due to less seed availability), and predicting the performance of traits that were not expressed in a particular environment owing to weather conditions (such as disease incidence) (Mohammadi et al., 2015; Millet et al., 2019; Cui et al., 2020; Krause et al., 2020).

Techniques like linkage and association mapping, marker-assisted selection (MAS), and GS need accurate phenotyping information for obtaining the desired results. GS requires phenotypic information for building models, and MAS requires phenotypic information for validating that a particular marker is associated with a trait (Kaur et al., 2021). In a large-scale breeding program, especially institutes such as the international maize and wheat improvement center (CIMMYT), international crops research institute for the semi-arid tropics (ICRISAT), international center for tropical agriculture (CIAT), and many breeding programs, approximately one hundred thousand breeding lines are screened every year at multiple locations, and the ability to accurately collect phenotyping data from this many lines and locations is challenging (Araus and Cairns, 2014; Araus et al., 2018; Zhang et al., 2020b; Juliana et al., 2020). Until now, advancements in phenotyping have not able to keep pace with developments in the field of genomics. However, the period from 2010 to 2019 witnessed the development and adoption of various phenomics tools in plant breeding under controlled and field conditions. Phenomics has unlocked the potential for phenotyping in plants for various traits like biotic (disease, insects, pests, viruses, and weeds) and abiotic stresses (drought, salinity, nutrient deficiency, flood, and other environmental factors), physiological (water use efficiency, photosynthesis mechanisms and different pigments), and agronomic traits (plant height, ear count and yield estimation) (Sankaran et al., 2015a; Zaman-Allah et al., 2015; Araus et al., 2018; Zhang et al., 2019). Merging phenomics with current genomics methods have improved progress in increasing the rate of genetic gain in many plant breeding programs (Masuka et al., 2017a, 2017b; Araus et al., 2018).

Several ground-based and aerial sensing platforms are being used with multiple sensors for measuring various traits in plants at different growth stages accurately, rapidly, and precisely (Sandhu et al., 2021e). The advancements in imaging sensors in plants varied from remote sensing to advanced autonomous vehicles equipped with RGB (red, green, and blue), near and far infrared, hyperspectral, light detection and ranging (LIDAR), 3D laser scanning, fluorescence, thermal, and spectro-radiometry imaging (Mewes et al., 2011; Atieno et al., 2017; Duan et al., 2018; Jimenez-Berni et al., 2018). Advanced autonomous platforms include ground robots, unmanned aerial vehicles (UAVs), and moving carts, which can take real-time data from several plots multiple times in a day to cover the whole season, generating enormous data for the plant breeders (Sankaran et al., 2019; Pattanashetti et al., 2020). Data generated from these sensors are longitudinally distributed in time and space, thus requiring skills from mathematics, statistics, data science, and machine learning for obtaining useful results, which could be merged with the genomic datasets and field breeding notes to make the best selections (Sun et al., 2017b; Sun et al., 2019).

The main objectives of this review are to 1) provide current status and overview about the advancements in genomics and phenomics for rice, wheat, soybean, common bean (*Phaseolus vulgaris* L.), chickpea (*Cicer arietinum* L.), and groundnut (*Arachis hypogaea* L). These six crops are chosen after considering the different rate of development during the last decade and importance in the human diet and crops were chosen separately from each cereal, legume and oilseed category; 2) offer an overview of GS and its implementation in cereal, legume, and oilseed breeding programs; 3) present developments in phenotyping platforms and imaging sensors for collecting phenotypic data; 4) discuss the status of below ground phenotyping techniques in plant breeding; and 5) discuss the merging of GS, machine learning, and phenomics information for increasing the genetic gain of breeding programs. This review is unique as it combines GS and phenomics in several important crops and will assist upcoming plant breeders understand the progress of this technology.

## Overview of Six Crops Used in This Study

**FIGURE 1:**
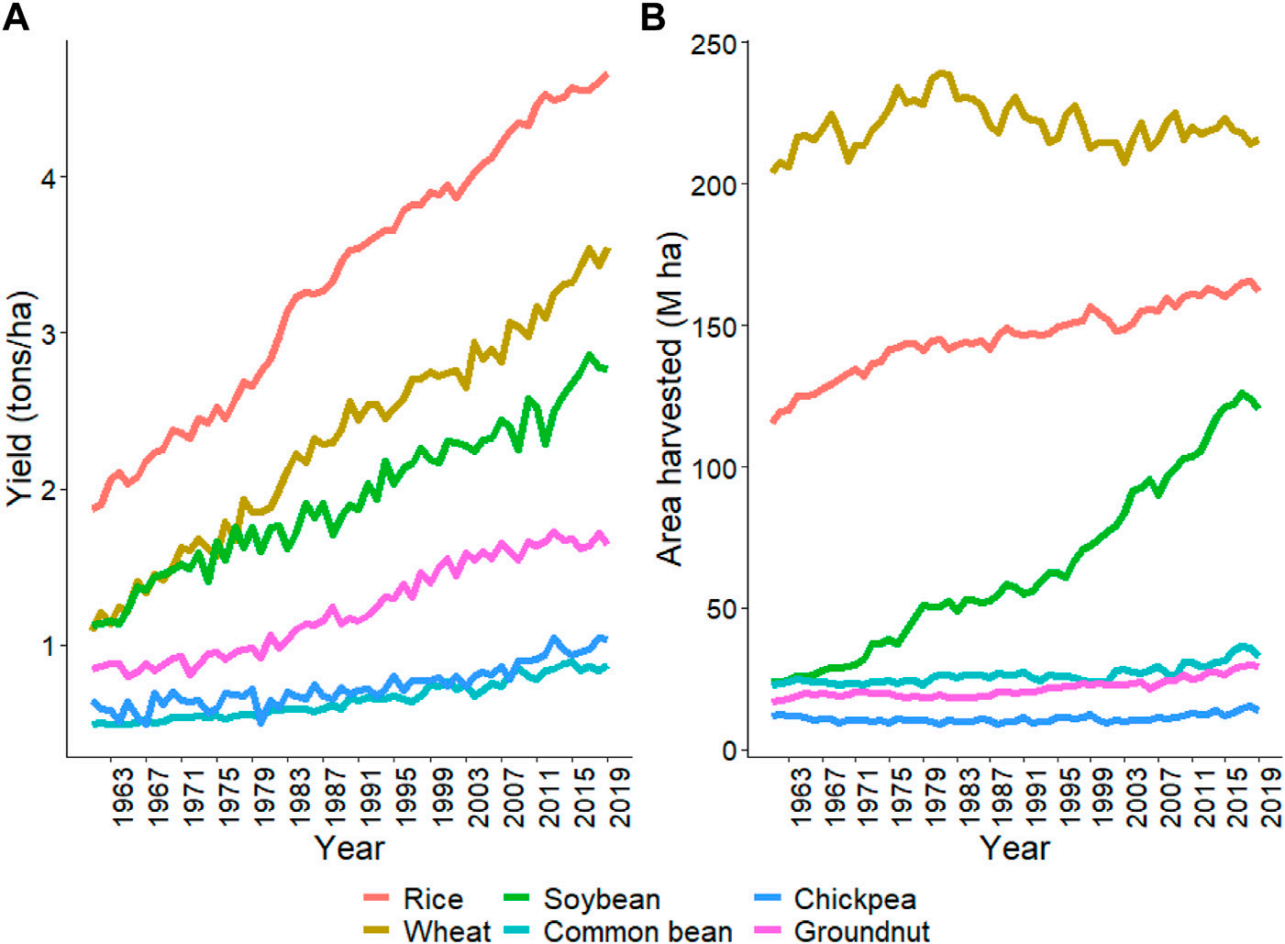
The trend for yield and area harvested for the six crops, namely, rice, wheat, soybean, common bean, chickpea, and groundnut, staring from 1961 to 2019. **(A)** shows the yield trend and **(B)** shows the total area harvested for each crop since 1961. Source FAO, 2019 dated 02/20/2021.

**FIGURE 2:**
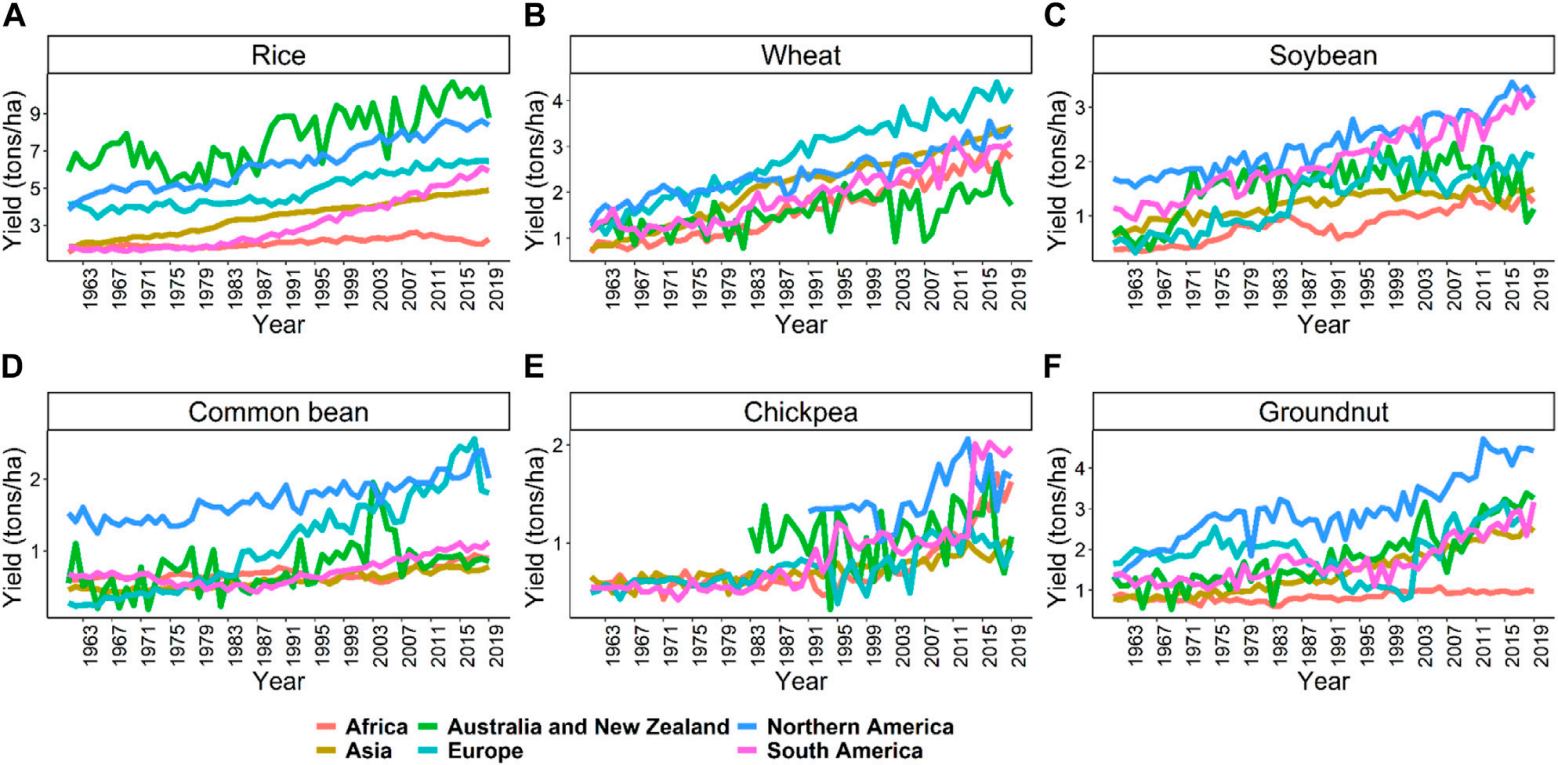
The average productivity of the six crops, namely, rice **(A)**, wheat **(B)**, soybean **(C)**, common bean **(D)**, chickpea **(E)**, and groundnut **(F)**, across the continents starting from 1961 to 2019. These trends show huge potential for improving the crops using genomics and high throughput phenomics approaches in the coming years. Source FAO, 2019 dated 02/20/2021.

This review focuses on six important crops: rice, wheat, soybean, common bean, chickpea, and groundnut, as described above. Average productivity and area harvested from these crops are provided in Figure 1 from 1961 to 2019 (FAO 2019) (https://www.fao.org/statistics/en/). The average productivity increased from 1.9 to 4.7 ton/ha in rice, 1.1–3.5 ton/ha in wheat, 1.1–2.8 ton/ha in soybean, 0.5–0.9 ton/ha in common bean, 0.6–1.0 ton/ha in chickpea, and 0.8–1.6 ton/ha in groundnut from 1961 to 2019 (Figure 1A). There was an approximately three-fold increase in rice, wheat, and soybean productivity due to breeding and agronomic efforts. However, in common bean, chickpea, and groundnut, similar gains have not been observed (Figure 1A). Total area harvested for rice, wheat, and soybean constantly increased from 1961 to 2019 compared to common bean, chickpea, and groundnut (Figure 1B). Organizations like CIMMYT, ICRISAT, and CIAT are working on collaborative projects to increase the crop’s yield and awareness among farmers to use better agronomic practices in these crops (Pandey et al., 2020b; Thudi et al., 2020). Figure 2 shows the productivity of these six crops across continents from 1961 to 2019. The green revolution has resulted in the highest increase in productivity of rice and wheat in Asia, but since the last 2 decades, the rate of increase is linear, which won’t be sufficient for the current increasing population, thus, demanding additional scientific and technological breakthroughs (Ray et al., 2013).

**FIGURE 3:**
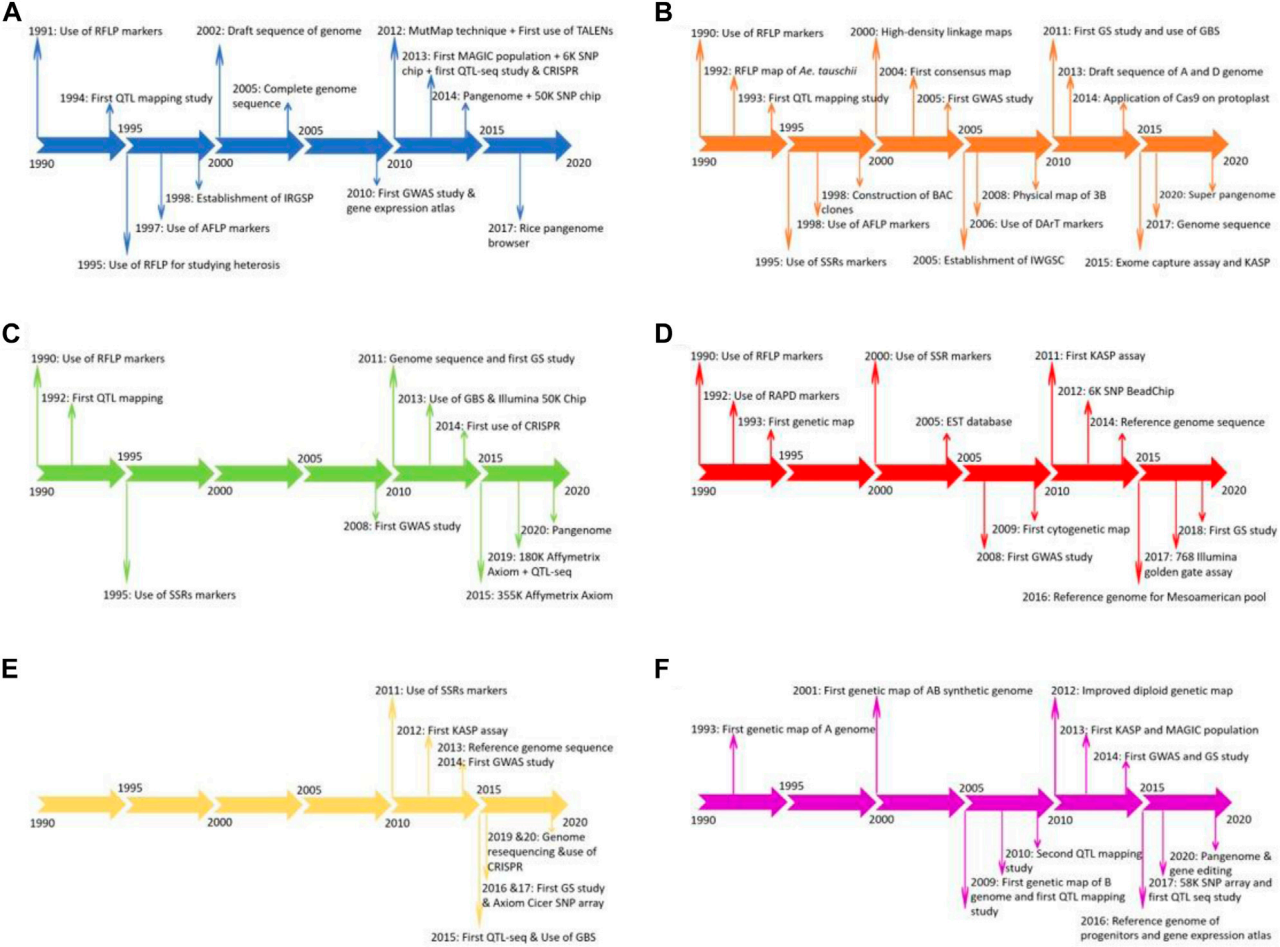
Timeline for advancement of genomics in rice **(A)**, wheat **(B)**, soybean **(C)**, common bean **(D)**, chickpea **(E)**, and groundnut **(F)**.

Rice is a major staple food consumed by more than one third of the world’s populations and meets up to 80% of the daily calorie intake for a vast majority of the Asian population (Kearney, 2010). Rice is a diploid species and has the smallest genome among the crops of economic importance, which assisted in its genome sequence in early 2002 (Sun et al., 2017a). Currently, several landraces, cultivar’s and wild relatives of rice have been sequenced, providing novel insights into the genome evolution of the crop and enhancing knowledge of new genes for rice breeding programs (Sun et al., 2017a). Due to its ease of transformation, abundant genetic and genomic resources (including mutants, cultivated landraces, and wild species), compact genome, and collinearity with other cereal crops, rice has become a model plant for crop genetic studies (Chen et al., 2014; Sun et al., 2017a). Rice was one of the crops which benefited from next generation sequencing due to its relatively modest level of repetitive sequences, making it easy to accurately align small reads to its reference genome (Abe et al., 2012; Takagi et al., 2013). Great success has been seen in rice for releasing cultivars having disease resistance, stress tolerance, improved nutritional value, and higher yield using CRISPR and other genome editing tools compared to the other five crops studied in this review (Mishra et al., 2018). The individual timeline for the genomics breakthrough in rice are depicted in Figure 3A.

Wheat is one of the three most consumed cereal crops globally, providing one-fifth of the total caloric input. It is grown on approximately 200 M ha globally and has widespread adaptation from 45 S in Argentina to 67 N in Scandinavia, including some high-altitude regions in the tropics and subtropics. Wheat went through two green revolution events, one in the late 1960s and another during the 1980s. During these green revolutions, the amount of gain for grain yield was approximately 3% in Asia, but has now declined to <0.9% annually, causing concern for breeders (Pingali, 2012). In spite of its hexaploid nature (2*n* = 6x = 42), wheat is one of the most widely studied crops at the genetic and cytogenetic level (Chhabra et al., 2021). The hexaploid nature of wheat has allowed the creation of major numerical and structural changes in chromosome constitution, that was made possible due to the efforts of Ernie Sears Sears et al, (1993). Sears et al. Sears et al, (1993) created aneuploid stocks of wheat, which were later used for several mapping and genome sequence studies. The last 3 decades witnessed a profound improvement in understanding wheat genomics and genetics due to the rapid adoption of DNA-based molecular markers such as RFLP, SSR, AFLP, DArT and SNPs from the early 1990s (Saini et al., 2022). These molecular markers have aided in conducting several QTL mapping studies using interval mapping, single-marker analysis, and GWAS (Muhu-Din Ahmed et al., 2020). Several development events in wheat, such as the first QTL mapping study, map-based cloning, first consensus map, adoption of high throughput genotyping arrays, translational genomics, gene editing, GS, and pangenome sequence are listed in Figure 3B to compare the development of genomics among the six crops (Poland et al., 2012; Rutkoski et al., 2016; Montenegro et al., 2017). Recently, the wheat pangenome sequence was released, with an average of 128,656 genes in each cultivar used, providing insights into genomic assisted crop improvement (Montenegro et al., 2017; Khan et al., 2020).

Soybean is a unique legume and oilseed crop consumed by humans, livestock, and poultry worldwide, as it is a rich source of protein, oil, essential amino acids, and metabolizable energy. The total protein and oil content is important for soybean, as 60% of its value comes from its meal and the remaining 40% from its oil (Warrington et al., 2015). A minimum of 47.5% protein content is required in soybean meal to develop livestock and poultry properly (Hurburgh et al., 1990). Although the domestication of soybean started in Asia, it found a welcomed home in the United States and Brazil. Brazil led production in 2019 (37%), closely followed by the United States (28%), Argentina (16%) and China (5%) (http://soystats.com/). Advancement of genomics started after 2010 in soybean with the genome sequence of cultivated soybean variety Williams 82 (Wm82) in the United States (Schmutz et al., 2010). In addition to the genome sequence of Wm82, several other accessions/lines were sequenced by China and Japan. The genome sequence was the base point for developing millions of SNP markers and thousands of SSR markers (Deshmukh et al., 2014). The development of next-generation sequencing and complexity reduction methods, namely GBS, restriction site-associated DNA (RAD) sequencing, and reduced representation libraries (RRL), are being routinely used. Technology advances have resulted in the development of several SNP arrays such as Illumina Infinium BeadChip (50K), Affymetrix Axiom (355K), Illumina Infinium BeadChip (8K), and Affymetrix Axiom (180), with many more routinely used for genotyping soybean plant introduction lines (Xu et al., 2013; Deshmukh et al., 2014). Recently, whole-genome assemblies released from 26 different soybean varieties and lead to the structuring of the soybean pangenome and the sequences of previously cultivated lines in the United States, China, and Japan (Liu et al., 2020). QTLs have been mapped for many quality, biotic and abiotic stress, and agronomic traits in soybean using QTL mapping and GWAS (Merry et al., 2019; Qin et al., 2019; Ravelombola et al., 2020; Shook et al., 2021). The complete details about the adoption of various genomic tools is presented as a timeline in Figure 3C.

Common bean is an important cultivated legume crop consumed worldwide, especially in developing countries in the tropics. It’s seed is rich in protein and other micronutrients like zinc and iron and provides a cheap energy source to millions of people in Africa, South Asia, and Latin America, where per capita consumption can reach up to 65 kg annually (Keller et al., 2020). Until now, the main hindrance in reaching the maximum threshold in bean is challenging environmental conditions. The important biotic and abiotic stresses affecting their performance include drought, low phosphorus, and diseases. Drought and low phosphorus have resulted in up to 70 and 50% yield loss and are the main focus for the common bean breeding programs worldwide (Beebe et al., 2008). Another important breeding objective is to reduce cooking time, as it retains the minerals and proteins which usually get lost with long cooking time. Less cooking time also saves energy and time for other tasks (Diaz et al., 2021). Mesoamerican and Andean have been described as two gene pools in common bean, with greater diversity present in the Mesoamerican pool. More progress for improving yield, disease resistance, and quality traits is reported in the Mesoamerican pool, but moving of genes/QTLs from this pool to the Andean pool has been challenging, especially due to linkage drag and incompatibility (Schmutz et al., 2014). Furthermore, with the sequencing of 100 landraces and 60 wild relatives, it is confirmed that there were two independent domestication events for common bean (Schmutz et al., 2014). MTAs have been performed for different disease traits, quality attributes, and yield traits for both pools in different studies (Giovannoni et al., 1991; Berry et al., 2020; Diaz et al., 2021). The timeline for the adoption of several genomic tools in common bean is provided in Figure 3D.

Chickpea is an important food legume crop grown on 13.72 M ha in 55 countries globally, producing 14.25 M tons (FAO 2019). Chickpea can produce 3.0–4.0 tons/ha, but currently it is restricted to ∼1 ton/ha due to limited work on biotic and abiotic stresses (Roorkiwal et al., 2018b). Total production of chickpea increased from 1961 at a slow pace due to the use and reuse of limited germplasm/donor parents (Varshney et al., 2013). Important abiotic stresses include drought and heat, while biotic stresses include ascochyta blight (*Ascochyta rabiei*), collar rot (*Sclerotium rolfsii*), dry root rot (*Rhizoctonia bataticola*), botrytis grey mold (*Botrytis cinerea*), and fusarium wilt (*Fusarium oxysporum*) that reduce crop yield. Chickpea is a rich source of dietary protein, minerals, carbohydrates, and essential nutrients, thus has the potential for improving malnutrition problems in south Asia and sub-Saharan Africa, where it is mostly grown (Varshney et al., 2013; Pandey et al., 2016; Roorkiwal et al., 2018b). The last couple of years have witnessed the adoption of several whole-genome sequencing and resequencing projects for sequencing several cultivars and landraces to explore genetic diversity (Verma et al., 2015; Varshney et al., 2019). The adoption of these next-generation sequencing methods in this decade has witnessed a shift from maker-based genotyping to sequenced based genotyping of diversified germplasm and breeding lines (Jaganathan et al., 2015; Li et al., 2018b). The development of chickpea varieties is further strengthened by the adoption of GS and speed breeding methods. The timeline for adopting several genomic tools in chickpea is provided in Figure 3E for comparison with other crops.

Groundnut or peanut is a nutritious oilseed and legume crop grown on 29.5 M ha in more than 100 countries globally, with a total productivity of 48.8 tons during 2019. Africa (55%) and Asia (40.3%) together have more than 95% of the groundnut cultivation area, account for 31.5 and 59.6% of the total production, respectively (FAO 2019). All parts of groundnut are a nutrition source for humans and animals. Groundnut plays an important role in fighting malnutrition as 80% of its seed consists of nutritious fats and proteins; furthermore, the crop can improve soil fertility and break the disease cycle when grown under rotation with cereal crops (Pandey et al., 2020b). Previously, groundnut was used as an edible crop in western countries, while in Asia and Africa, it was mainly used for oil production. The development of high oleic acid groundnut lines and awareness about its nutritional value has resulted in the rapid adoption of this crop as a primary food source across the globe. Genomic studies in groundnut gained momentum after the first SSR based genetic map was developed in 2009 (Varshney et al., 2009). Several MAGIC and NAM populations were developed for deciphering the genetic architecture of complex traits like aflatoxin contamination, oleic acid content, drought, and disease tolerance (Pandey et al., 2016; Chu et al., 2018). The last decade was the golden era for developing genomics in groundnut and several resources, such as a reference genome for cultivated tetraploid and progenitors, high density genotyping, genome-wide genetic markers, gene expression atlases, and MAGIC and NAM populations, were developed, with a timeline shown in Figure 3F (Akohoue et al., 2020; Pandey et al., 2020b, 2020a). Still, this crop has many other priorities for coming years like reference genome sequence for wild diploids, functional genomics, and high throughput genotyping assays, which might improve breeding for groundnut.

## Genomic Selection and Its Implementation in the Breeding Program

**FIGURE 4:**
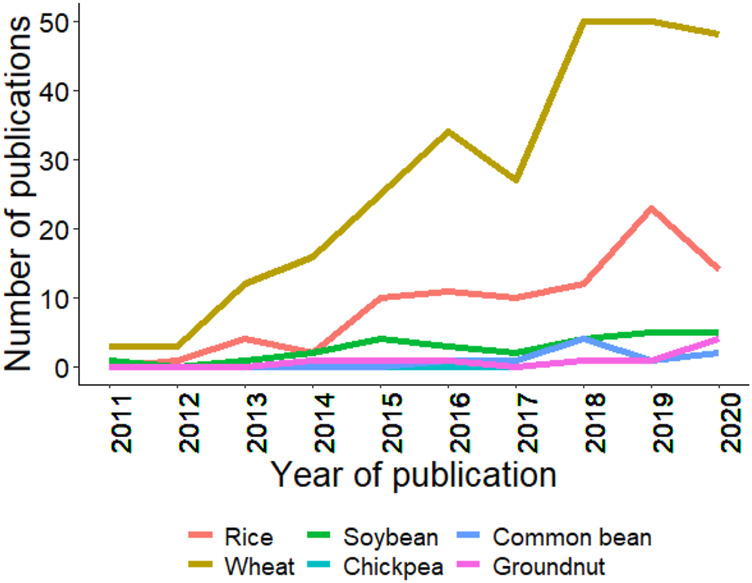
Trends in publications mentioning/discussing the six crops and genomic selection since last decade (2011–2020). Search was made using associated crop and genomic selection keywork in the abstract. Source PubMed dated 02/25/2021.

As mentioned in the introduction, GS is a technique for predicting GEBVs using training and testing populations (Bhandari et al., 2019; Crossa et al., 2019). GS has been efficiently applied in wheat, rice, and soybean; however, in crops like chickpea, common bean, and groundnut, its progress is slow. Figure 4 summarizes the trends for GS studies conducted from 2011–20, and it is clear that GS was rapidly adopted in wheat, and other crops are following the trend at a slower pace. The slow rate of adoption in chickpea, common bean, and groundnut is due to the recent advancement of genomics tools, genome sequences, assembly of the core collection, pangenome, and whole-genome resequencing (Verma et al., 2015; Roorkiwal et al., 2018a; Pandey et al., 2020b). Thus, the coming years will see efforts in the adoption of GS and other new genomics tools to improve the genetic gain for these globally important crops.

Several factors affect the performance of GS models. They have been explored in multiple studies during the last decade, ranging from training population size, relatedness between training and testing population, cross-validation strategy, marker density, heritability of the trait, population structure, and prediction model (Yabe et al., 2018; Frouin et al., 2019; Huang et al., 2019). It is observed that a certain population size is required for model training to avoid model overfitting (Liu et al., 2018). A large training population size results in higher prediction accuracy; however, a smaller than desirable size is often used due to the costs associated with their phenotyping and genotyping (Heffner et al., 2011). In wheat, it was observed that prediction accuracy constantly increased when training population size was increased from 24 to 96 (Heffner et al., 2011). Similarly, another study in wheat showed the same trend when population size was increased from 250 to 2000 (Heslot et al., 2012). Relatedness between genotypes in the training and testing sets significantly affects prediction accuracy (Lozada et al., 2019). More related lines share common ancestors in a small number of prior generations, have fewer recombination events, and conserve marker and QTL linkage phases (Heslot et al., 2012). The effect of training population size is not observed on prediction accuracy when individuals are closely related in the training and testing set (Mujibi et al., 2011).

Since GS uses genome-wide markers, proper genotyping is required. To date, several genotyping platforms like RFLP, AFLP, SSR, DArT, and SNP chips have been explored for GS; however, since 2012, with the emergence of the GBS platform, it has dominated all previous platforms due to the low cost, genome-wide coverage, and reduced sampling bias compared to SNP chips (Poland and Rife, 2012; Poland, 2015). It has been seen that large marker density results in model overfitting, causing lower independent prediction accuracies (Werner et al., 2018). However, larger marker density is favored as it increases the probability of LD between the QTL and marker. Lower LD combined with a larger training population and higher marker density largely improves prediction accuracy (Crossa et al., 2014; Norman et al., 2018). Heritability and population size plays an important role in prediction, as they determine the amount of genetic variation that the associated prediction model could capture (Guo et al., 2014). A strong correlation is observed between the GS model’s prediction accuracy and the trait’s heritability in the training population (Edwards et al., 2019). Various parametric and non-parametric machine and deep learning models have been explored for GS in all the mentioned crops (Table 1). Until now, none of the models have significantly demonstrated superiority for all traits in all crops (Liu et al., 2019; Ravelombola et al., 2020; Sandhu et al., 2021b). Breeders should explore various models in their programs for different traits and use the best performing model final predictions after considering accuracy, error and computational burden (Wang et al., 2018). Table 1 provides information about models explored for GS, with their associated characteristics and links to the source codes, for breeders, if they want to explore them in their crop of interest.

**TABLE 1:** The detailed information about various models explored for genomic selection in different crops, with associated model type, characteristics, and links to the source codes that could be easily implemented in various breeding programs.

Model	Model type	Characteristics	Codes	References
Ridge regression best linear unbiased prediction (RRBLUP)	Mixed model/parametric model	Ridge regression is equivalent to traditional BLUP, which assumes each marker has a small effect with constant variance and obtain this by using ridge regression parameter	https://github.com/cran/rrBLUP	Endelman (2011)
Genomic best linear unbiased prediction (GBLUP)	Mixed model/parametric model	GBLUP uses the relationship between genotypes for predicting their performance	https://github.com/gdlc/BGLR-R/blob/master/inst/md/GBLUP.md	Bernardo and Yu (2007)
Bayes A	Bayesian model/parametric model	Marker effects are obtained assuming a scaled inverted chi-square distribution of variance parameters, and all the markers are assumed to have an effect	https://github.com/gdlc/BGLR-R/blob/master/inst/md/Validation.md	Meuwissen et al. (2001)
Bayes B	Bayesian model/parametric model	It losses the restrictions of Bayes A and allows some markers to have zero effect	https://github.com/ShiuLab/GenomicSelection/blob/master/working/predict_BGLR.R	Meuwissen et al. (2001)
Bayes C	Bayesian model/parametric model	Bayes C uses the scaled-t mixture with a point mass at zero with scaled-t distribution	https://github.com/cma2015/G2P/blob/master/R/GSEnsemble.R	Pérez and De Los Campos (2014)
Bayes Cpi	Bayesian model/parametric model	Bayes Cpi is a special case of Bayes B but it assumes a constant variance for markers with non-zero effect	https://github.com/gdlc/BGLR-R/blob/master/inst/md/BayesianAlphabet.md	Pérez and De Los Campos (2014)
Bayes D	Bayesian model/parametric model	Bayes D uses the scaled-t distribution by estimating scale parameter from the datasets	https://github.com/gdlc/BGLR-R/blob/master/inst/md/BayesianAlphabet.md	Pérez and De Los Campos (2014)
Bayes Lasso (BL)	Bayesian model/parametric model	BL assumes a fixed set of markers have zero effect, and the remaining follow the double exponential distribution for variance components	https://github.com/Sandhu-WSU/Genomic-Selection-tutorial/blob/master/GSworkshop_InProg.R	Tishbirani (1996)
Elastic net (EN)	Parametric model	EN is the intermediate between ridge regression and Lasso using an average weight penalty for marker effect estimation	https://datadryad.org/stash/dataset/doi:10.5061/dryad.7f138	Crain et al. (2018)
Bayesian threshold GBLUP (TGBLUP)	Bayesian model/parametric model	TGBLUP is a threshold models for ordinal and categorical data	https://github.com/gdlc/BGLR-R	Montesinos-López et al. (2019a)
Bayesian multi-trait and multi-environment model (BMTME)	Bayesian model/parametric model	BMTME is the multi-trait version of the Bayesian models	https://www.g3journal.org/content/9/5/1355#app-1	Montesinos-López et al. (2018)
Reproducing kernel Hilbert space (RKHS)	Bayesian kernel-based/semi-parametric model	RKHS uses the kernel functions on the set of distances among markers to estimate the relationship matrix between the individuals and assumes the absence of linearity assumption	https://github.com/gdlc/BGLR-R/blob/master/inst/md/RKHS.md	Pérez and De Los Campos (2014)
Reaction norm	Mixed model/parametric model	Reaction norm model the interaction between the markers and environmental covariates using covariate functions	https://github.com/gdlc/BGLR-R/blob/master/inst/md/BayesianAlphabet.md	Jarquín et al. (2014)
Support vector machine (SVM)	Machine learning/semi-parametric model	SVM is another semiparametric model that uses kernel function, and its cost function is sensitive to residuals coefficient	https://github.com/afiliot/Kernel-Methods-For-Genomics	Ravelombola et al. (2020)
Random forests (RF)	Machine learning/non-parametric model	RF uses a network of the tree with varying number of nodes, mtry, and depth for building the final forest for predictions	https://github.com/xuanxu/nimbus	Guo et al. (2020a)
Gradient boost machine (GBM)	Machine learning/non-parametric model	GBM is an ensemble learning model and is similar to tree-based models used to reduce the subset the SNPs using linkage disequilibrium for obtaining higher prediction accuracy	https://cran.r-project.org/web/packages/gbm/index.html	Li et al. (2018a)
Functional B spline	Machine learning/non-parametric model	Functional B splines use the piecewise polynomial of degree n-1 in a variable x. Different spline functions are tried at a given degree for predicting the output		Montesinos-López et al. (2017)
Partial least square regression (PLSR)	Machine learning/non-parametric model	PLSR is a dimensional reduction approach which uses latent variables derived from predictors to link with the response variables	https://datadryad.org/stash/dataset/doi:10.5061/dryad.7f138	Crain et al. (2018)
Multilayer perceptron (MLP)	Deep learning/non-parametric model	MLP uses the combination of input, multiple hidden and output layers using a large number of neurons for building the relationship between the predictors and output	https://github.com/saeedkhaki92/Yield-Prediction-DNN	Sandhu et al. (2021b)
Convolutional neural network (CNN)	Deep learning/non-parametric model	CNN employs convolutional, flattening, pooling, and dense layer for prediction using filers and kernels to reduce the excess predictors from the model	https://github.com/Sandhu-WSU/DL_Wheat	Sandhu et al. (2021b)
Dual CNN	Deep learning/non-parametric model	Dual CNN uses two parallel streams of CNN and sums up layer is used for predictions	https://github.com/kateyliu/DL_gwas	Liu et al. (2019)
Arc-cosine kernel (AK)	Deep learning/non-parametric model	AK estimates the stepwise covariance matrix by adding more hidden layer in model training	https://www.frontiersin.org/articles/10.3389/fgene.2019.01168/full#h7	Crossa et al. (2019)
DeepGS	Deep learning/non-parametric model	DeepGS uses deep CNN consisting of one input, one convolutional, one sampling, two fully connected and one output layer for building a relationship	https://github.com/cma2015/DeepGS	Ma et al. (2018)
Recurrent neural network (RNN)	Deep learning/non-parametric model	RNN is best for predictions under the presence of longitudinal or time-series data, as it uses the memory state to retains the information from previous data and update its prediction with new information	https://figshare.com/s/5cd5e5e4eaeef55b721f?file=24963563	Maldonado et al. (2020)

**FIGURE 5:**
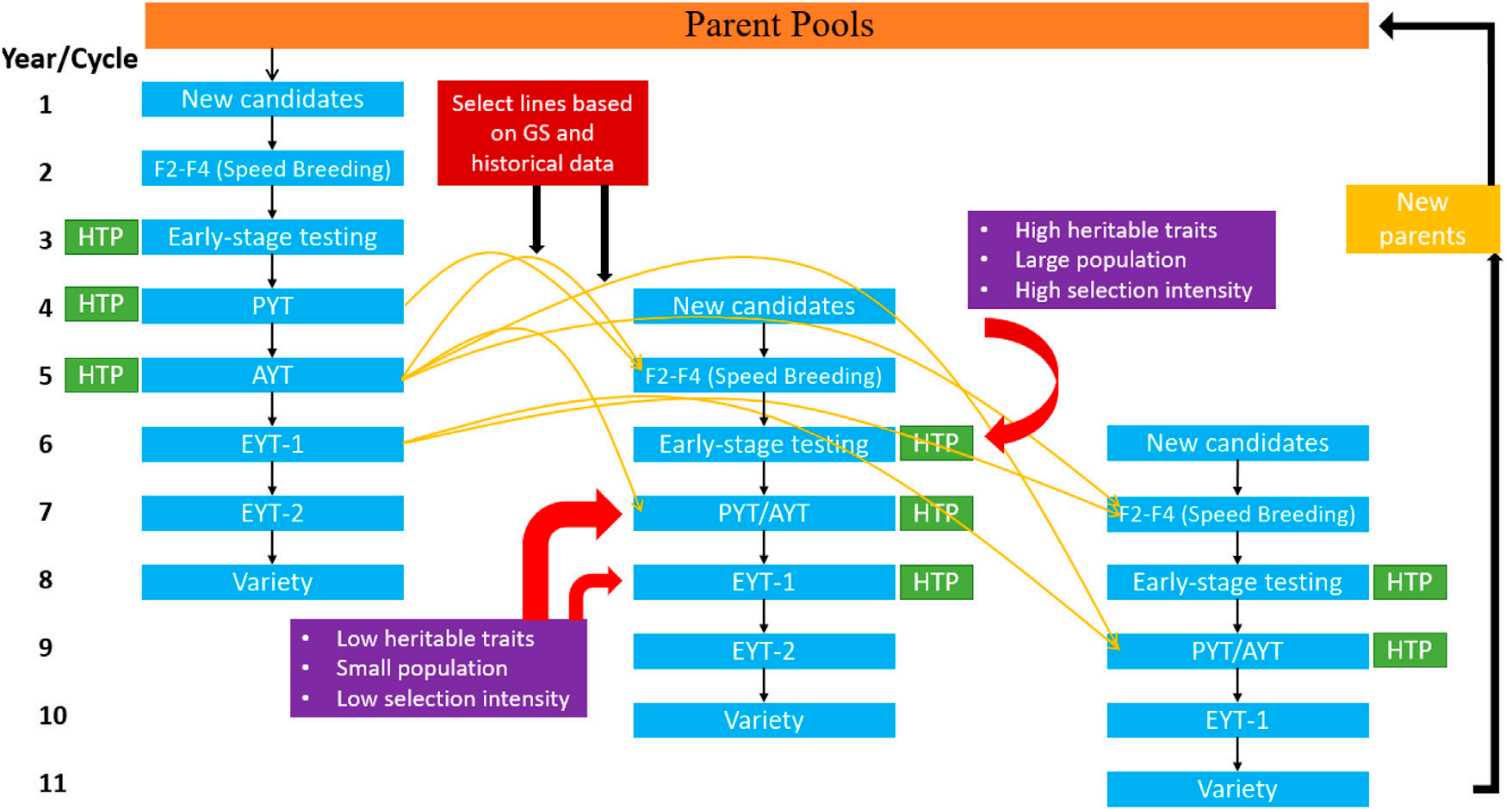
The standard breeding scheme outline for self-pollinating crops with the implementation of genomic selection and phenomics information for predicting various traits earlier in the pipeline in different selection cycles. Three columns show the three separate breeding cycles starting from the cross initiation to the variety release. The yellow arrows represent how genomic selection can be used on datasets from previous years to predict phenotype in F2-F4 stage and early-stage testing stages. The red arrows show the stages where selection is imposed for low and high heritable traits in traditional breeding; however, with genomic selection, decisions can be performed for low heritable traits earlier in the pipeline. Here it is assumed that a single generation is planted in a year. The DH represents the double haploid, PYT is preliminary yield trial, AYT is advanced yield trial, and EYT is elite yield trial.

GS is being applied with two approaches in the plant breeding program. Firstly, it is applied at the early generation (F_1_) or (F_2:3_) for a rapid generation cycle with a short interval. This selection is used to predict the breeding values and helps the researchers select parents for new crosses or remove inferior performing lines earlier in the pipeline (Bassi et al., 2015; Gaynor et al., 2017). Therefore, linear additive models are sufficient for predicting at this stage. The second approach involves predicting the plant’s total genetic value by considering additive, dominance, epistasis, and environmental effects (Monteverde et al., 2019; Francki et al., 2020; Guo et al., 2020b). Genetic values are predicted for most environments using different combinations of environment, genotype by environment, and weather parameters in the GS models (Monteverde et al., 2019; Francki et al., 2020). Rapid progress is happening in the second approach for predicting traits in an untested environment with better prediction accuracy (Jarquín et al., 2017; Gill et al., 2021) (Table 2). We provided an outline of the implementation of GS in a plant breeding program for self-pollinated crops, where GS could be either applied within the cycle selection, across cycles, with multi-location selection and the inclusion of genotype by environment interactions, and utilization of phenomics datasets for improving prediction accuracies for complex traits (Figure 5). In this outline, it is assumed that a single generation is possible in a year until speed breeding is used to reach homozygosity (Watson et al., 2018).

**TABLE 2:** Genomic selection studies covering important breeding traits conducted in 2019 and 2020 for rice, wheat, soybean, chickpea, common bean, and groundnut throughout the world. Complete detail about the population size, validation strategy, marker density, model type, the accuracy obtained and country where the study was conducted is provided.

Crop	Trait	Population size	Validation strategy	Marker intensity	Model	Single or multi-trait analysis	Accuracy	Country	References
Rice	Arsenic content	228 accessions	CV and IV	22,370 SNPs	GBLUP, Bayes A, RKHS	Single trait	0.43–0.48	France	Frouin et al. (2019)
	Days to heading	112 cultivars	CV and IV	408,372 SNPs	GBLUP	Single and multi-trait models	0.93–0.98	Japan	Jarquin et al. (2020)
	Drought tolerance	280 accessions	CV	215,000 SNPs	GBLUP and RKHS	Single and multi-environment models	0.22–0.80	France	Bhandari et al. (2019)
	Grain weight distribution	128 cultivars	CV	42,508 SNPs	GBLUP and PLS	Single trait models	0.28–0.53	Japan	Yabe et al. (2018)
	Grain yield and quality traits	327 & 320 breedinglines	CV and IV	92,430 and 44,598 SNPs	GBLUP, PLS, and reaction norm mode	Single and multi-environment models	0.11–0.82	Uruguay	Monteverde et al. (2019)
	Rice blast	161 and 162 accessions	CV and IV	66,109 and 29,030 SNPs	GBLUP, Bayes A, Bayes C and MLP	Single and multi-trait models	0.15–0.72	United States	Huang et al. (2019)
	Ten agronomic traits	1,495 hybrid rice	CV and IV	232,935 SNPs	GBLUP, additive and dominance model	Single trait	0.54–0.92	China	Cui et al. (2020)
Wheat	Anther extrusion	603 lines	CV and IV	7,649 SNPs	Reaction norm model	Single trait models	-0.03–0.74	CIMMYT	Adhikari et al. (2020)
	Days to heading	286 accessions	CV	9,047 SNPs	RRBLUP, BA, BB, BC, BL, and BRR	Single trait models	−0.04–0.45	Iran	Shabannejad et al. (2020)
	Days to heading and plant height	3,486 lines	CV	2,083 SNPs	MTDL	Multi-trait models	0.39–0.62	CIMMYT	Montesinos-López et al. (2019b)
	End-use quality traits	401 lines	CV and IV	4,598 SNPs	RRBLUP	Single trait models	0.38–0.63	Austria	Michel et al. (2018)
	End-use quality traits	1912 lines	CV and IV	21,210	GBLUP	Single and multi-trait models	0.28–0.69	France	Ben-Sadoun et al. (2020)
	End-use quality traits	179 lines	CV	16,383 SNPs	RRBLUP	Single trait models	0.10–0.48	Spain	Mérida-García et al. (2019)
	Fusarium head blight and Septoria tritici blotch	642 lines	CV and IV	8,398 SNPs	RRBLUP	Single trait models	−0.41–0.88	Germany	Herter et al. (2019)
	Grain yield	1,325 lines	CV and IV	9,290 SNPs	GBLUP	Single and multi-trait models	0.18–0.31	Denmark	Tsai et al. (2020)
	Grain yield	1716 lines	CV and IV	15,853 SNPs	RRBLUP	Single trait	−0.05–0.20	United States of America	Lozada et al. (2020b)
	Grain yield, days to heading, and plant height	270 lines	CV and IV	14,163 SNPs	GBLUP and DL	Single and multi-environment models	0.02–0.91	CIMMYT	Montesinos-López et al. (2019c)
	Grain yield and end-use quality traits	282 lines	CV and IV	7,426 SNPs	BL, RF, RKHS, and RRBLUP	Single trait	0.07–0.68	United States	Hu et al. (2019)
	Grain yield and protein content	480 lines	CV	7,300 SNPs	GBLUP	Single and multi-trait models	−0.60–0.74	Austria	Michel et al. (2019a)
	Leaf and stripe rust	1744 single crosses	CV	15K SNPs	GBLUP	Single trait	0.16–0.50	Germany	Beukert et al. (2020)
	Powdery mildew	467 lines	CV	34,095 SNPs	RRBLUP	Single trait model	0.36–0.67	United States of America	Sarinelli et al. (2019)
	Septoria tritici blotch	175 lines	CV	6,097 SNPs	RRBLUP	Single trait model	0.47–0.62	Sweden	Odilbekov et al. (2019)
	Snow mold	753 lines	CV and IV	12,681 SNPs	RRBLUP, GBLUP and RKHS	Single trait	−0.09–0.92	United States	Lozada et al. (2019)
	Winter hardiness and frost tolerance	504 lines	CV and IV	1,413 SNPs	GBLUP	Single traits	−0.02–0.58	Austria	Michel et al. (2019b)
Soybean	Amino acids	249 lines	CV	23,279 SNPs	RRBLUP and GBLUP	Single trait model	0.18–0.85	United States	Qin et al. (2019)
	Chlorophyll content tolerance	172 lines	CV	4,089 SNPs	RRBLUP, GBLUP, BL, RF and SVM	Single trait model	0.31–0.76	United States	Ravelombola et al. (2019)
	Soybean cyst nematode	234 lines	CV	3,782 SNPs	RRBLUP, GBLUP, BL, RF and SVM	Single trait model	0.05–0.53	United States	Ravelombola et al. (2020)
	Yield, protein content, oil and height	5,000 lines	CV	4,236 SNPs	DualCNN, deepGS, singleCNN and RRBLUP	Single trait model	0.23–0.47	United States	Liu et al. (2019)
	Yield	5,600 lines	CV	4,600 SNPs	GBLUP	Single trait model	0.27–0.60	United States	Howard and Jarquin (2019)
Groundnut	Leaflet length, 100 seed weight, days to maturity and total yield	281 accessions	CV	493 SNPs	RRBLUP	Single trait model	0.02–0.62	South Africa	Akohoue et al. (2020)
	Seed weight, oleic acid content, total yield, and days to maturity	340 lines	CV and IV	13,355 SNPs	Reaction norm model	Single and multi-environment models	0.19–0.89	India	Pandey et al. (2020a)
Chickpea	Grain yield, podding time, emergence score and seed number	132 lines	CV	144,777 SNPs	BL and BRR	Single trait model	0.22–0.81	Australia	Li et al. (2018b)
	Seed weight, biomass, harvest index, and seed yield	320 breeding lines	CV and IV	89,000 SNPs	Reaction norm models	Single and multi-environment models	−0.01–0.94	India	Roorkiwal et al. (2018b)
Common bean	Cooking time, and water absorption capacity	922 lines	CV and IV	5,738 SNPs	RKHS, BA, BC and BL	Single trait model	0.22–0.55	Colombia	Diaz et al. (2021)
	Grain yield and days to maturity	481 breeding lines	CV and IV	5,820 SNPs	GBLUP, BL, BA, BB, and RKHS	Single and multi-environment models	0.6–0.8	Colombia	Keller et al. (2020)

Figure 5 provides the outline for a breeding cycle for wheat and few modifications can be made in this scheme in order to adjust for other crops. In the first year two different parents are crossed with subsequent chromosome doubling in the second year using double haploid (DH) or any other technique for reaching 100% homozygosity (i.e., speed breeding, single seed descent, rapid generation advance, shuttle breeding or tissue culture). These early stage testing lines are evaluated in the third year, and selection is made for high heritable traits, like pod type in groundnut and soybean cyst nematode resistance (Akohoue et al., 2020; Ravelombola et al., 2020). Each set of early-stage testing progenies has a specific set of genes, and the breeder aims to identify the best combination for advancing to the next generation and seed multiplication trial. The measurement of several agronomic traits, such as grain yield and aflatoxin content in groundnut, quality attributes in rice, common bean and wheat, for which a large amount of seed is required, is not possible at this stage (Battenfield et al., 2016; Pandey et al., 2020a). Seeds from the selected lines are multiplied at a single location known as a preliminary yield trial (PYT), and spectral information could be collected using phenomics tools like unmanned aerial vehicles (UAVs), remote sensing, handheld scanners, or tractor-mounted instruments (Rutkoski et al., 2016; Sandhu et al., 2021c). The information generated with these phenomics tools provides a secondary source of trait information for selecting complex traits by understanding G by E interaction, field variation, and explanation of various physiological processes occurring in the plants. Furthermore, these phenomics tools have been used to measure several agronomic traits and disease severity more efficiently and effectively. The lines selected from the PYT are later planted for 1 year at various locations with different replications depending upon the seed generated in the PYT and constitutes the advanced yield trials (AYT). Spectral information can be collected in a similar way as done during PYT to increase selection efficiency. After AYT, breeders keep reducing the population’s size, owing to limited resources and space, and selected lines are continually planted at multiple locations for measuring more quantitative traits. This step is repeated for two-three years depending upon the trait and constitutes elite yield trials (EYT) (Bassi et al., 2015; Gaynor et al., 2017).

Across cycles, predictions are possible at early stages, when seed is limited, to measure quantitative traits like grain yield, end-use quality traits in rice and wheat, and protein content in chickpea and common bean (Jernigan et al., 2018; Diaz et al., 2021). Figure 5 shows that GS and phenomics data sets collected at PYT and AYT from the previous cycle could be used to predict quantitative traits for the F2-F4 population and early-stage testing lines in a new selection cycle. Similarly, in the subsequent years, data from previous cycles and the same cycle can predict AYT performance at multiple locations (Montesinos-López et al., 2017; Crain et al., 2018). Phenomics information provides a significant advantage for within cycle and across cycle prediction in multi-trait GS models. Spectral reflectance indices (SRI) derived from these phenomics measurements have increased prediction accuracy in various GS studies in wheat (Rutkoski et al., 2016; Crain et al., 2018; Sandhu et al., 2021c). Higher prediction accuracies are obtained for grain yield due to lower heritability and higher genetic correlation with SRI. Utilization of these SRI in multi-trait GS models, and as a covariate in the GS models, increases the capture of total variation for a particular trait and helps explain various physiological phenomena that are difficult to observe under field conditions (Rutkoski et al., 2016; Lozada and Carter, 2019). We were not able to find any GS study which used phenomics information in GS models in chickpea, common bean, and groundnut. Table 3 provides the studies that have used GS and phenomics information for predictions in wheat, and the improvement in the model’s performances are provided. There is a significant advantage of including phenomics datasets in GS models due to observed increase in prediction accuracy, suggesting that merging these two techniques can assist in increasing the yield of these crops in the coming decade.

**TABLE 3:** Genomic selection studies that have used phenomics information in wheat is summarized. The traits or spectral information derived from the phenomics data sets and the physiological parameters which they explain is provided with information about their effect on the final prediction accuracies when included in the genomic selection model is added to show their potential.

Trait	Population size	Model	Phenomics traits used	Physiological trait explained	Effect on prediction accuracy with inclusion of phenomic information	Country/institute	References
Grain yield	1,092 lines	RRBLUP	GNDVI, RNDVI and CT	Canopy size, greenness, chlorophyll content, and temperature	70% increase in prediction accuracy	CIMMYT	Rutkoski et al. (2016)
Grain yield	1,092 lines	GBLUP, EN, and PLSR	CT and NDVI	Canopy temperature and greenness	7% increase in prediction accuracy	United States	Crain et al. (2018)
Grain yield	3,282 lines	RRBLUP	CT and GNDVI	Canopy temperature and nitrogen status	46% increase in prediction accuracy	United States	Sun et al. (2019)
Grain yield	456 lines	Recommender system & GBLUP	NDVI, NWI, and SR	Biomass, greenness, and water status	19% increase in prediction accuracy	United States	Lozada and Carter (2020)
Grain protein content	650 lines	RRBLUP	NDVI, GNDVI, NWI, WI, ARI, and PRI	Biomass, chlorophyll, nitrogen, water, anthocyanin, and photochemical pigments status	20% increase in prediction accuracy	United States	Sandhu et al. (2021c)
Grain yield	4,368 lines	GBLUP	GNDVI	Biomass and greenhouse	11–23% increase in prediction accuracy	CIMMYT	Juliana et al. (2019)
Grain yield	242 lines	GBLUP	CT, SPAD, SGT, NDVI	Canopy temperature, chlorophyll content, stay green and senescence traits	63% increase in prediction accuracy	United States	Guo et al. (2020b)
Grain yield	1716	RRBLUP	NDRE, NDVI, and SR	Biomass, vegetation, and water status	13% increase in prediction accuracy	United States	Lozada et al. (2020a)
Grain yield	771 lines	GBLUP	Reflectance bands	Whole spectrum from visible to infra-red were used	10–16% increase in prediction accuracy	CIMMYT	Krause et al. (2019)
Grain yield	630 lines	Random regression and GBLUP	CT and NDVI	Canopy temperature and biomass	70% increase in prediction accuracy	United States	Sun et al. (2017b)

## Development in Phenotyping Platforms and Imaging Sensors

The last three-decades witnessed an unprecedented increase in the adoption and development of genomics in plant breeding programs, leading to a rise in genetic advances in the major cereal crops (Thudi et al., 2020). However, genetic gain has stagnated in major cereal crops globally, which requires the need to raise the efficiency of breeding programs. It is perceived that limitations in the progress and development of phenotyping tools and platforms contribute to lower efficiency in breeding (Rincent et al., 2018). With this in mind, several phenomic initiatives and facilities have been launched at regional, national, and international levels; still, breeders are skeptical about the application of these tools (Atieno et al., 2017; Duan et al., 2018). Breeders are concerned that results obtained from phenotyping platforms under controlled conditions are not indicative of field performance for complex traits, especially under large environmental variability (Atieno et al., 2017; Duan et al., 2018). Moreover, the high throughput platform’s extensive phenotyping is onerous and not cost-efficient compared to the benefits achieved so far. Lastly, data generated from these tools results in data management and big data problems, causing an issue for making a legitimate conclusion for decision-making without understanding data science and machine learning models (Singh et al., 2016). In spite of these challenges, several phenomics platforms, tools, and sensors have been developed, and their improvement and adoption rate is fairly high with the hope of breaking this stagnated genetic advance (Ashourloo et al., 2014; Dobbels and Lorenz, 2019). The next one or 2 decades have considerable potential for phenomics to reach the stage where genomics is today, allowing collection of a large amount of data, gaining understanding from previously unknown traits, and making valid conclusions based on those.

Imagers and sensors have allowed collection of multidimensional and high-resolution datasets from plants to quantify crop growth, yield, biotic or abiotic stress, and other physiological processes under both fields and controlled conditions (Cai et al., 2016; Sankaran et al., 2019). These sensors can measure spectral reflectance ranging from radio waves to gamma waves of the electromagnetic spectrum and create an abundance of information to select from. The resulting imaging sensors varies from LIDAR, X-ray computed tomography (CT), time-of-flight based systems, positron emission tomography, thermal, visible to near-infrared, multispectral, hyperspectral, fluorescence, and stereovision (Kobayashi et al., 2001; Zhang et al., 2018). The field-based platforms range from Internet-of-Things (IoT) based sensor systems, field mounted system (e.g., tower), tractor/sprayer modified systems (manually operated), small autonomous systems, scanning platforms, UAVs, aircraft, and more recently, low orbiting satellite systems (Sangjan et al., 2021). In general, most of the phenotyping systems in controlled environment are commercial systems developed by the private industry. Recently, there has been interest in the development of IoT based systems for customized operation in controlled environment (Sangjan et al., 2021). The commonly used sensors in the phenotyping platforms used in plant breeding are RGB, multispectral, hyperspectral, thermal, and fluorescence sensors employed on ground-based or aerial platforms. These can cover large numbers of plots at a time by measuring absorption, reflection, and refraction information from the plant canopy. RGB sensors are most often used owing to their cost and simplicity (Ashourloo et al., 2014). All these remote sensing tools provide information about several physiological parameters related to crop yield by considering the plant’s nutrient, water, radiation, pigment contents, resource allocation, and biomass partition (Duan et al., 2018; Dobbels and Lorenz, 2019). Most imagers and sensors are equipped on ground-based platforms, mainly stationary in the field or on phenomobiles at experimental facilities to develop new applications and require specialized training and considerations for their use (Cai et al., 2016; Jimenez-Berni et al., 2018). The increase in resolution and miniaturization has lowered their cost and could be easily purchased by small scale labs. The main success in plant phenotyping has come with higher resolution and miniaturization of the sensors coupled with UAVs for covering a large number of plots in a limited time frame and is preferred over the ground-based platforms in many programs (Sankaran et al., 2015b; Gracia-Romero et al., 2019).

**FIGURE 6:**
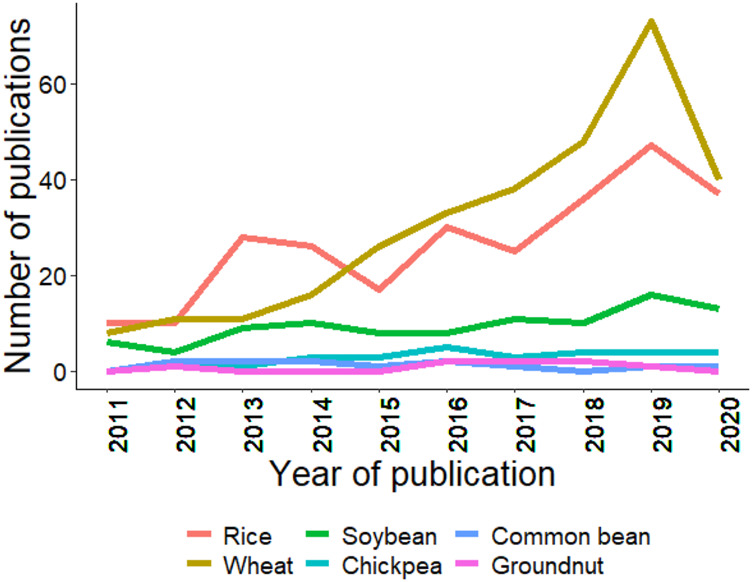
Trends in publications mentioning/discussing the six crops and high throughput phenotyping since last decade (2011–2020). Search was conducted using associated crop and high throughput phenotyping keywords in the abstract. Source PubMed dated 02/20/2021.

Figure 6 provides the studies using high throughput phenotyping (HTP) in these six crops for the last decade (2011–2020). An observed 3–4 fold increase in the number of studies that are using HTP for rice and wheat can be found, but for chickpea, common bean, groundnut, and soybean, there is no improvement observed in this regard (Zhang et al., 2020a; Zhang et al., 2021). Fewer number of studies using HTP in chickpea, common bean, and groundnut might be attributed to the recent adoption of genomics technology (Pandey et al., 2020b). These crops can still benefit from the use of HTP technology to better evaluate various agronomic, biotic, and abiotic stress-related traits. Table 4 shows recent studies conducted for these six crops where different phenotyping platforms and imaging sensors were used for various agronomic, biotic, and abiotic stress studies. In general, most of the studies used RGB or multispectral imaging due to their lower cost, easy management of data, and avoidance of problems related to big data. Furthermore, UAVs have relatively high adoption rates over ground-based platforms by utilizing the same imaging sensors with better resolution and throughput in collecting data from large plots.

**TABLE 4:** Important studies conducted using phenomic tools in the last decade for the six crops explored in this study. Information about the trait, phenotyping platform, sensor and study description is provided.

Crop	Trait	Platform	Sensor/imager	Discrimination	References
Rice	Rice blast	Hand-held, simulating aircraft imagery	Multispectral imaging	Reflectance values in the visible and near-infrared regions were used to link with a disease severity rating	Kobayashi et al. (2001)
	Rice sheath blight	UAVs	RBG and multispectral imaging	Derived vegetation indices from multispectral images and percentage of infected leaf areas with RGB were used for disease detection	Zhang et al. (2018)
	Drought stress	Greenhouse automated system at the Rice Automatic Phenotyping (RAP) facility in Germany	RGB imaging	Stay green values were used to assess the stress tolerance ability of genotypes	Duan et al. (2018)
Wheat	Powdery mildew	German Aerospace Centre	Hyperspectral imaging	Powdery mildew was detected, and the best hyperspectral bands were identified for detecting this fungal disease for application in breeding programs	Mewes et al. (2011)
	Leaf rust	Hand-held ground-based sensing	RGB imaging and multispectral (spectroradiometer) sensors	Vegetation indices from multispectral imaging and percentage of infected leaves from RGB imaging were used for the classification of leaf rust	Ashourloo et al. (2014)
	Plant biomass and height	Phenomobile portable buggy	3D imaging with LIDAR	Plant height, biomass, and canopy cover was measured in a labor-intensive way	Jimenez-Berni et al. (2018)
Soybean	Seed yield	UAVs	RGB imaging	Average canopy cover obtained at an earlier stage was used as a covariate in yield prediction models	Moreira et al. (2019)
	Iron deficiency	UAVs	Multispectral imaging	Image processing and unsupervised classification models were used for classifying the iron-deficient plots	Dobbels and Lorenz (2019)
	Seed yield	UAVs	Hyperspectral imaging	Feature selection approach was used to identify best bands for predicting seed yield with different ML models	Yoosefzadeh-Najafabadi et al. (2021)
Chickpea	Salinity tolerance	Plant accelerator installed at University of Adelaide	RGB imaging	The plant growth rate was monitored throughout the growth stages to study the effect of salinity	Atieno et al. (2017)
	Progression of senescence	Camera established on a stand	RGB imaging	Color distortion correction algorithms were applied on time series data to quantify the onset and progression of senescence	Cai et al. (2016)
Common bean	Seed yield and biomass	UAVs	Multispectral imaging	Derived vegetation indices showed a strong relationship with seed yield and biomass	Sankaran et al. (2019)
	Root architecture	Root excavation, ground-based	RGB imaging and traits estimation with DIRT	Genotypes were differentiated for their root traits	Burridge et al. (2016)
Groundnut	Iron deficiency	Chlorophyll meter SPAD, Hand-held	Infrared sensor	Genetic loci associated with increasing iron deficiency were identified	Pattanashetti et al. (2020)

RGB and multispectral imaging have shown a tremendous adoption rate during the last decade for studying biotic and abiotic stresses in crops. Rice sheath blight (*Rhizoctonia solani*) and blast (*Magnaporthe oryzae*) are devastating diseases of rice observed worldwide, and accurate detection and management are the focus of several breeding programs. RGB and multispectral imaging sensors on UAVs are an affordable and user-friendly option for disease detection and rating (Kobayashi et al., 2001; Zhang et al., 2018). Color space transformation and color feature extraction have been used to select the diseased varieties or qualitatively detect the infected portions; however, estimation of disease quantitatively was less effective. Vegetation indices extracted from multispectral images showed high accuracy for quantitatively predicting these diseases (Kobayashi et al., 2001; Zhang et al., 2018). Hyperspectral imaging covers a broader region of the electromagnetic spectrum (400–2,500 nm) with a narrow bandwidth, non-destructively explaining various biochemical and physiological changes occurring in the plant due to environmental conditions. For example, in wheat, hyperspectral imaging has been used to detect powdery mildew severity and infection using feature selection algorithms (Mewes et al., 2011). As hyperspectral imaging provides information about various spectral bands, most of which are unnecessary, feature selection is required. This became possible due to the adoption of machine learning models by plant breeders. Here, Mewes et al. (2011) used support vector machine and spectral angle mapper classification methods for feature selection to identify the most important spectral band. Later, those selected bands showed higher prediction accuracy for powdery mildew.

Phenomics aids in the collection of high-quality data earlier in the breeding pipeline from thousands of breeding plots with high temporal and spatial resolution (Krause et al., 2020). Data collected at earlier stages in the growth cycle has shown an advantage in soybean breeding, where canopy coverage during vegetative growth stages have high heritability and genetic correlation with seed yield (Moreira et al., 2019). UAVs are commonly used for collecting canopy coverage with RGB cameras, which is later used for predicting seed yield from multiple plots (Moreira et al., 2019). In a recent study, Yoosefzadeh-Najafabadi et al. (2021) used hyperspectral imaging collected at vegetative stages in soybean and feature selection with machine learning models and demonstrated 93% prediction accuracy for seed yield prediction. There are various other examples where phenomics is used in soybean for studying biotic stresses (powdery mildew, phomopsis seed decay, and target spot), abiotic stresses (nutrient deficiency, drought, and waterlogging), and agronomic traits (seed yield, pod number and biomass estimation) (Mo et al., 2015; Moreira et al., 2019; Yoosefzadeh-Najafabadi et al., 2021). Multiple vegetation indices [normalized difference vegetation index (NDVI), normalized water index (NWI), photochemical reflectance index (PRI)] derived from multispectral imaging were used to find the best time point for predicting the above ground mass and seed yield using correlation and regression analysis (Sankaran et al., 2019). Furthermore, thermal sensors were used to obtain the mean plot temperature and showed a high correlation with plant biomass (Sankaran et al., 2019). A couple of studies have shown the potential of multispectral imaging using UAVs for common bean to predict the seed yield and biomass, but the total number studies are limited when compared to wheat, rice, and soybean (Figure 6) (Burridge et al., 2016; Sankaran et al., 2019).

Ascochyta blight is a devastating disease in chickpea, and remote sensing has shown opportunities for its monitoring in the field (Zhang et al., 2019). Multispectral and thermal sensors deployed on UAVs were used to extract canopy area, percentage of cover, and vegetation indices for predicting disease severity and seed yield in chickpea. The study showed the potential for timely management of the disease by monitoring the crop with remote sensing techniques (Zhang et al., 2019). In a different study, two hundred forty-five chickpea accessions were evaluated using image-based phenotyping to study genetic variation for salt tolerance (Atieno et al., 2017). Pod abortion and pod filling inhibition are the main effects of salinity, and imaging sensors were used to identify the accessions with salt tolerance by phenotyping pod number and seed density (Atieno et al., 2017). In groundnut, iron deficiency occurs when plants are grown on neutral and alkaline soils, reducing the availability of Fe^2+^ in plants. Infrared sensors were used in groundnut for measuring chlorophyll and iron deficiency chlorosis systems (Pattanashetti et al., 2020). The adoption of phenomics for groundnut in high production countries like India and Ethiopia offers an advantage for reducing yield gaps by understanding various physiological and biochemical process, along with genomic technologies, to improve yield performance.

## Going Underground, a Challenge for a Breeder

**FIGURE 7:**
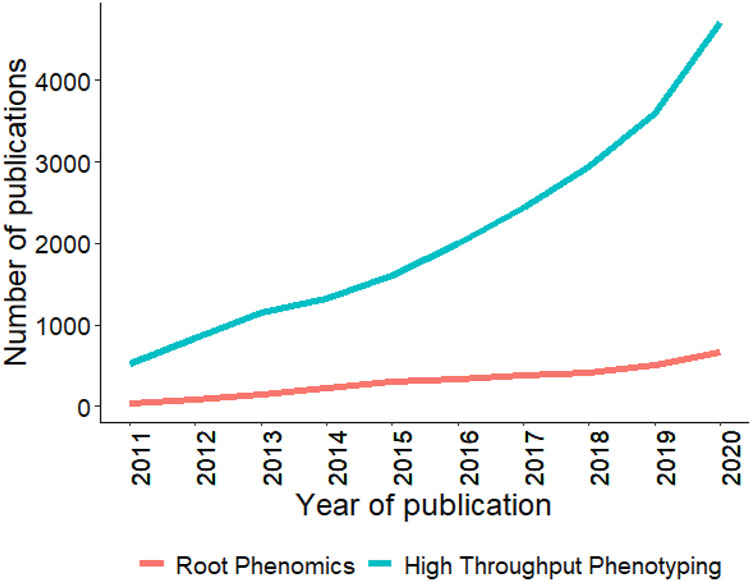
Trends in publications mentioning/discussing root phenomics and high throughput phenotyping since last decade (2011–2020). Search was conducted using root phenomics and high throughput phenotyping keywords in the abstract. Source PubMed dated 02/20/2021.

Although genomics and phenomics tools have helped plant breeders study above-ground traits in great detail, limited work has been done on belowground root systems, which play a vital part in a plants affecting overall grain yield potential. Figure 7 shows the trend for publications using HTP and root phenomics, and it can be concluded that root phenotyping studies are lagging behind other phenomics studies. Roots play an essential role by directly influencing plant growth by regulating water and nutrient uptake, regulating drought stress, resisting soil-borne diseases, and maintaining the crop’s yield and quality (Seck et al., 2020; Wu et al., 2021). The study of root system architecture (RSA) is challenging *in situ* compared to above-ground phenotyping. Several 2D transparent growth mediums are available that allow sequential capturing of RGB imaging to study growth dynamics and root hair development, such as PlaRoM, Rhizoslides, RootPainter, SNAP, Rhizovision, Rhizoponics, RADIX, and RhizoTubes (Le Marié et al., 2014; Mathieu et al., 2015; Falk et al., 2020; Smith et al., 2020). Various open-source image analysis tools like SmartRoot, RootNav, RootTrace, and EZ-Root-VIS are available to study RSA (French et al., 2009; Pound et al., 2013). To study 3D RSA, a gel-based cylinder can be used to study up to 16 roots traits (Iyer-Pascuzzi et al., 2010). Other 3D image reconstruction and image analysis tools are RootReader3D and GiaRoots (Iyer-Pascuzzi et al., 2010). All these platforms work under lab conditions.

The above-mentioned transparent media does not entirely mimic field conditions. GROWSCREEN-Rhizo, an intelligent mechanized root phenotyping platform, was developed to phenotype roots and shoots simultaneously in transparent soil-filled rhizotrons (Bodner et al., 2018). In a separate study, these Rhizotrons were equipped with thermal and hyperspectral cameras for measuring the temperature and root chemical components like lignin change, water content, and mineral observation capacity (Pound et al., 2013; Le Marié et al., 2014). The difference in the X-ray attenuation capacity of roots and soils is utilized to visualize the inner 3D structure in the X-ray CT. Open-source tools like RootViz3D and RooTrak are used for analyzing different X-ray attenuation capacity to reconstruct the 3D RSA (Mairhofer et al., 2015). However, X-ray CT suffers from some limitations, which vary from the impact of soil type, compaction, and homogeneity of soil particles on X-ray attenuation values. Furthermore, high doses of X-ray affect plant and microbial growth in the soil, and lastly, scanning resolution and volume increase the time of data collection for large pots, limiting the frequency of data acquisition (Metzner et al., 2015).

Other root phenotyping approaches include positron emission tomography (PET), magnetic resonance imaging (MRI), thermal neutron tomography, and neutron radiography. MRI uses the absorption and re-emission of electromagnetic radiation from the nuclei to determine its root architecture and functional attributes (Courtois et al., 2013; Beyer et al., 2019). But MRI is highly sensitive to moisture content and is only applicable if the root diameter is more than 1 mm. Similarly, PET uses the radiotracer distribution for non-invasively studying root attributes. PET has been used to scan the roots up to 85 mm deep non-invasively, and used to monitor carbohydrate transportation assimilates over a more extended period (Garbout et al., 2012). X-ray CT, MRI, and PET have been used differently and have their own strengths and limitations, and hence used interchangeably. For instance, 1) PET has lower signal deterioration by water content and soil structure compared to CT and MRI; furthermore, high water content affects the performance of CT more than MRI (Garbout et al., 2012); 2) CT is more effective for providing high-resolution information from small pots; however, when pot size is large, MRI provides more information about root structure than CT (Pflugfelder et al., 2017); 3) MRI and CT provide higher spatial resolution than PET, but PET provides better contrast between roots and soil owing to gamma radiation; and 4) PET and MRI scanning requires a large amount of time compared to CT, and is problematic for genetic studies where a large number of samples need to be screened (Metzner et al., 2015). In regard to the *in-situ* root phenotyping in field conditions, there has been great interest in utilizing ground penetrating radar (GPR) (Atkinson et al., 2019). But similar to other techniques, there are limitations associated with influence of soil type and condition on data quality. Table 5 provides information about various other root phenotyping techniques. Advancements in root phenotyping in recent years shows the potential for improving below ground traits in all the crop species by understanding traits better. Further reading about the below-ground phenotyping can be found in other review articles (Paez-Garcia et al., 2015; Wasaya et al., 2018).

**TABLE 5:** Description of the important root phenotyping techniques and associated growth media’s for studying the root system architecture.

Root phenotyping technique	Growth media	Description	References
Shovelomics	Soil (field based)	Involved excavation of root samples from the soils to visually score various attributes. The pipeline involves digging of sample, soaking and rinsing, picture collection and finally scoring the characteristics	Garbout et al. (2012)
Digital imaging	Liquid media (lab)	Roots are scanned in a liquid media for length, diameter, topology, and branching patterns	Piñeros et al. (2016)
Digital imaging	Growth pouch system	Roots are scanned in a growth pouch medium for length, diameter, topology, and branching patterns	Falk et al. (2020)
Soil coring	Soil (field based)	It uses tractor mounted hydraulic soil corer for digging steel alloy sampling tubes into soil and assist in phenotyping roots	Iyer-Pascuzzi et al. (2010)
Minirhizotrons	Soil (field-based)	A transparent tube is permanently inserted into the ground and growth of shoot and root is continuously monitored throughout the growth stages	Le Marié et al. (2014)
Rhizolysimeters	Soil (field-based)	It uses underground concrete pipes, silos and corridor to house soil containing cores for constant observation of root traits	Bodner et al. (2018)
Rhizoponics	Liquid media (lab)	It is combination of rhizotrons and hydroponics, where set up is immersed in tank filled with media. Non-destructive 2D imaging of roots and shoots is performed	Mathieu et al. (2015)
X-ray CT	Soil (greenhouse and lab)	X-ray CT non-destructively measures the attenuating ionizing radiations for assessing the root structure and constructing the 3D image of RSA	Metzner et al. (2015)
Ground penetrating radar	Soil (field-based)	It is mostly used for tree roots and uses electromagnetic pulse system for determining root diameter, biomass, and other attributes	Garbout et al. (2012)
Positron emission tomography	Liquid media (lab)	It uses the functional and molecular imaging for tracing the radio tracer distribution in the plant non-invasively	Garbout et al. (2012)
Magnetic resonance imaging	Soil (greenhouse and lab)	This study the magnetic moment of atomic particles using strong magnetic and radio frequency	Pflugfelder et al. (2017)

Developing crop varieties which remain productive on marginal soils and under water deficit is the main aim of several breeding programs, especially in Asia and Africa, owing to climate change (Pattanashetti et al., 2020). Breeding programs maintain yield by selecting combinations of traits like increased harvest index, increased shoot biomass, resistance against insects and pests, and altering the duration of the growing season (Mathieu et al., 2015; Atieno et al., 2017). However, these traits might be linked to root traits, but are not explored to such an extent. This could be achieved using several root phenotyping techniques under field, greenhouse, and laboratory conditions (Iyer-Pascuzzi et al., 2010). Various QTLs were identified controlling RSA for assistance in genomic assisted breeding (Li et al., 2017; Zhao et al., 2019; Seck et al., 2020). QTLs were identified controlling root branching, root length, root hair, and other root traits in certain crops. Identification of QTLs or genes controlling these traits requires accurate and reproducible phenotyping information (Li et al., 2017; Seck et al., 2020). Although several QTLs have been identified for these RSA traits, information is still lacking, such as their mechanism, effect under different genetic backgrounds, and role under different environments and soil types. Most of the roots traits identified so far are polygenic and demonstrate a tremendous potential for utilization of GS for predicting RSA by building reliable training sets for the crops (Li et al., 2017; Seck et al., 2020).

In a recent study, two hundred wheat lines were screened for root dry matter, root diameter, seminal axis root length, root dry matter, and branching pattern in seedling growth over the hydroponic system for performing MTAs (Beyer et al., 2019). From this study, 63 QTLs were identified to control these RSA traits and have a minor effect on phenotypes, suggesting the polygenic nature of these five traits in wheat (Beyer et al., 2019). A root phenotyping study was conducted on 529 rice accessions under controlled and drought conditions to identify MTAs for 21 traits. Researchers identified 264 QTLs controlling all 21 traits, and most of them were already reported in previous studies in rice, further validating the genetic architecture of root traits (Courtois et al., 2013; Li et al., 2017). Similarly, in soybean, GWAS has been performed in various studies to explore RSA trait’s genetic architecture. A recent study using 137 soybean lines grown under rhizoboxes and phenotyped with two-dimensional imaging identified 10 QTLs controlling 15–20% variation for primary root diameter and total root length (Seck et al., 2020). As common bean is mainly grown under drought conditions, 196 QTLs were identified in 438 accessions for various root traits such as root length/weight, lateral root length, taproot length, root volume, root surface area, average root diameter, and lateral root number under drought conditions (Wu et al., 2021). This study provided the genetic basis for roots traits under drought conditions, which will ultimately improve common bean (Wu et al., 2021). There was no major finding related to the study of genetic architecture for RSA traits for chickpea and groundnut, providing opportunities for adoption of root phenotyping in the coming years. We were also not able to find any study using GS for predicting root traits. This will be an emerging research area in coming decades due to rapid progress in root phenotyping that will help understand the genetic architecture of root traits, creating datasets for training GS models, and ultimately helping the breeder select multiple traits simultaneously.

## Merging of Genomic Selection, Phenomics and Machine Learning in Breeding

As discussed previously, GS aids in predicting GEBVs and in increasing genetic gain by reducing variety development time and cost per cycle and increasing selection accuracy. Phenomics allows generation of high-quality quantitative data and effectively characterizes large breeding populations (Araus et al., 2018). It has been seen that there is potential for combining GS and phenomics for increasing efficiency and precision while minimizing labor and lowering costs. This will aid in increasing the selection intensity and accuracy within breeding programs and subsequently the selection response (Sun et al., 2017b; Sandhu et al., 2021c). Until now, data from phenomics tools have been used as secondary traits for evaluating disease and pest resistance, abiotic stresses, end-use quality traits, and ultimately grain yield. Furthermore, phenomics datasets are collected in a longitudinal framework that helps select individuals with a specific spectral trajectory during a particular growing stage and helps predict temporal breeding values for specific periods (Moreira et al., 2020). Table 3 provides most of the studies that have used phenomics datasets in multi-trait GS models to predict grain yield in wheat and observed the improvement in the prediction accuracy, either by using single indices or multiple indices in the models.

Most of the GS studies conducted so far use a single trait (univariate) statistical model to predict one trait at a time and do not benefit from the genetic correlation among two or more traits (Jia and Jannink, 2012; Galán et al., 2020). However, multi-trait (multivariate) GS has demonstrated increased prediction accuracy, reduced selection trait bias, high statistical power, and increased parameter estimation accuracy (Sandhu et al., 2021a). Multi-trait GS models have more advantages for traits with low heritability values, like grain yield and end-use quality traits, where secondary traits correlated with high heritability values aid in increasing prediction accuracy (Crain et al., 2018; Lozada and Carter, 2019; Sandhu et al., 2021c). Recently, several studies from CIMMYT have demonstrated an increase in prediction accuracy for grain yield in wheat when secondary longitudinal data collected from phenomics is included as a covariate or in multi-trait GS models (Sun et al., 2019; Lozada et al., 2020a). Furthermore, secondary traits extracted from phenomics aid in selecting earlier in plant growth stages for quantitative traits, allowing earlier program resource allocation to the best individuals. In addition to increasing prediction accuracy, selection response, and intensity, longitudinal phenomics data can explain the various biological process underlying plant growth, not limited to water status, biomass accumulation, chlorophyll content, and photosynthetic efficiency. Primarily SRI are extracted from these longitudinal phenomics data which indirectly explain important physiological processes and stresses in the plants and are mainly used in multi-trait GS models.

Rutkoski et al. (2016) used SRI extracted from phenomics datasets and included them into pedigree and GS models for predicting grain yield in wheat. Doing this in earlier stages of the breeding pipeline is advantageous to remove poorly performing lines, but GS is sometimes not possible at this stage owing to genotyping cost. They showed that pedigree information could also be used with SRI for predicting grain yield earlier to enhance genetic gain. Pedigree information removed the cost and effort of genotyping a large number of plants, and their use also satisfies Mendelian sampling. Rutkoski et al. (2016) observed a 56 and 70% improvement in prediction accuracy for grain yield for within environment predictions using pedigree and genomic relationship matrices when including SRI in the models. The indices used in the study were canopy temperature and green normalized difference vegetation index (GNDVI), which provided information about canopy temperature and biomass and were phenotypically and genetically correlated to grain yield (Rutkoski et al., 2016). In another study, Sun et al. (2017a) used NDVI and canopy temperature in a multi-trait, random regression, and repeatability model for predicting grain yield in wheat and observed a 70% increase in prediction accuracy compared to the single trait GS model. Furthermore, the multi-trait model’s average improvement in predictability was highest, followed by random regression and repeatability model. Various other studies obtained similar results by the inclusion of secondary traits in wheat (Sun et al., 2017b; Crain et al., 2018).

Campbell et al. (2018) used longitudinal phenomics data for fitting random regression models to predict shoot growth trajectories in rice using pedigree and genomic relationships by fitting a second-order Legendre polynomial. A random regression model with longitudinal phenomics data demonstrated improvement in prediction compared to a single data point in traditional mixed linear models. They also showed the future growth predictions could be performed with high accuracy by using a genomic random regression model by having a subset of early phenomics measurements (Campbell et al., 2018). Similarly, another study in rice used random regression models by fitting B-spline and second-order Legendre polynomials to predict the projected shoot area under water-limited and controlled conditions and demonstrated that random regression models performed better than the baseline multi-trait models (Campbell et al., 2019). Furthermore, B-spline models fit a better curve compared to Legendre polynomials (Campbell et al., 2019). Therefore, we have seen that predominantly random regression models are used in rice for fitting or predicting growth curves. In contrast, in the case of wheat, multi-trait GS models have shown the advantage to predict quantitative traits using longitudinal phenomics datasets, which results in significant improvements compared to traditional models. Additional studies suggest canopy cover plays an important role in predicting the seed yield for soybean. Jarquin et al. (2018) modeled the genetic covariance between canopy cover collected by phenomics tools and seed yield using various cross-validation schemes and molecular markers to predict the seed yield. The prediction ability was highest when both canopy cover and molecular markers were included in the prediction models compared to only molecular marker and canopy information (Jarquin et al., 2018).

Owing to the ability of phenomics to collect a large amount of data due to its high spatial and temporal variation, it sometimes creates the big data problem, where feature selection needs to be performed, and complex machine and deep learning models are needed to build the relationship between features and predictors. Several machine and deep learning models, such as random forests, ensemble-based methods, support vector machine, multilayer perceptron, convolutional neural network, and recurrent neural network, are often employed for analyzing phenomics data and predicting traits with GS models. The main interests for these models in plant breeding are useful due to their powerful ability to learn the complex/hidden non-linear relationship in the data to predict complex traits and usually result in higher prediction accuracy than a mixed linear GS model. Ma et al. (2018) and Sandhu et al. (2021e) have shown the potential of deep learning models for predicting grain yield in wheat and observed higher prediction accuracies than the previous BLUP based models and open up a new class of models that could be explored. Table 1 provides the various machine and deep learning models, and their source code links, which have been explored for GS so far. In the coming years, an active area of research is merging machine and deep learning models with phenomics datasets and molecular markers to predict the breeding program’s complex traits.

## Concluding Remarks

We explored six important self-pollinated crops consumed by 90% of the world population. Most of the advancements in genomics and phenomics over the last decade have been observed in wheat and rice. The genome sequencing of other crops and the adoption of high throughput genotyping tools have paved the way for understanding various underlying genetic mechanisms. These crops can utilize phenomics in coming years after seeing the progress and benefits achieved in wheat and rice. Several GS models varying from traditional BLUP based model to machine/deep learning models have been explored for prediction. Furthermore, the inclusion of genotype by environment interaction in these models has delivered good prediction accuracy for predicting untested lines in new environments. All the GS models discussed in this study, including genotype and environment interaction, will assist the plant breeder in making improved selection decisions. Multi-trait GS models also indicate their success for predicting low heritable traits and will be explored in future years for prediction under multi-environment scenarios, with the inclusion of phenomics datasets, for understanding genotype by environment interactions.

The yield trends of crops across different continents is very diverse, and it is evident that in Asia and Africa, use of advanced genomic and phenomic technologies can improve/enhance grain yield. Furthermore, public breeding programs play a predominant role in these crops. To translate the advantage of GS and phenomics in their programs, low-cost genotyping and phenotyping needs to be developed and used. In this context, easy to handle, reliable, and affordable low throughput platforms pave the way, and among such tools, RGB cameras make good candidates. Below ground phenotyping is tedious for a plant breeder and is being ignored by most programs. However, several field and lab-based root phenotyping tools were launched in the last decade and their potential is being realized. Further refinement and throughput will pave a new way to better understand root traits in field crops. This is extremely important for continuously increasing drought, salinity, aluminium, and heavy metal sensitivity to plants. As the total number of studies for phenotyping the roots traits increase, this will ultimately aid in predicting new genotypes using GS once enough data are collected for each crop under the different breeding programs.

This review highlights the advantages of combining genomics and phenomics, especially in wheat and rice. There is a need to merge and adopt these two disciplines at a fast pace in other crops to increase their genetic gain. GS has been shown to increase genetic gain by increasing selection accuracy and intensity with reduction of cycle time, which can be further enhanced by using phenomics, and machine/deep learning models in the breeding programs due to big data sets. These tools could aid in screening large number of lines with less phenotyping cost and efforts, allowing better exploration of the genetic diversity of particular crops for various traits. Phenomics is assisting plant breeders in integrating physiological breeding in addition to using molecular and genetic tools for selection. Thus, future studies in breeding will focus on merging all these tools and domains to reach the required rate of genetic gain for grain yield.
